# Pitavastatin Reduces Inflammation in Atherosclerotic Plaques in Apolipoprotein E-Deficient Mice with Late Stage Renal Disease

**DOI:** 10.1371/journal.pone.0138047

**Published:** 2015-09-14

**Authors:** Manabu Shibasaki, Jian-Guo Wang, Jose-Luiz Figueiredo, Sophie E. P. New, Thibaut Quillard, Claudia Goettsch, Jun-ichiro Koga, Hiroyuki Sonoki, Jiro Matsumoto, Masanori Aikawa, Elena Aikawa

**Affiliations:** 1 Center for Interdisciplinary Cardiovascular Sciences (CICS), Division of Cardiovascular Medicine, Brigham and Women’s Hospital, Harvard Medical School, Boston, Massachusetts, 02115, United States of America; 2 Center for Excellence in Vascular Biology, Division of Cardiovascular Medicine, Brigham and Women’s Hospital, Harvard Medical School, Boston, Massachusetts, 02114, United States of America; Innsbruck Medical University, AUSTRIA

## Abstract

**Objectives:**

Chronic renal disease (CRD) accelerates atherosclerosis and cardiovascular calcification. Statins reduce low-density lipoprotein-cholesterol levels in patients with CRD, however, the benefits of statins on cardiovascular disease in CRD remain unclear. This study has determined the effects of pitavastatin, the newest statin, on arterial inflammation and calcification in atherogenic mice with CRD.

**Methods and Results:**

CRD was induced by 5/6 nephrectomy in cholesterol-fed apolipoprotein E-deficient mice. Mice were randomized into three groups: control mice, CRD mice, and CRD mice treated with pitavastatin. Ultrasonography showed that pitavastatin treatment significantly attenuated luminal stenosis in brachiocephalic arteries of CRD mice. Near-infrared molecular imaging and correlative Mac3 immunostaining demonstrated a significant reduction in macrophage accumulation in pitavastatin-treated CRD mice. Pitavastatin treatment reduced levels of osteopontin in plasma and atherosclerotic lesions in CRD mice, but did not produce a significant reduction in calcification in atherosclerotic plaques as assesses by histology. CRD mice had significantly higher levels of phosphate in plasma than did control mice, which did not change by pitavastatin. In vitro, pitavastatin suppressed the expression of osteopontin in peritoneal macrophages stimulated with phosphate or calcium/phosphate in concentrations similar to those found in human patients with CRD.

**Conclusion:**

Our study provides in vivo evidence that pitavastatin reduces inflammation within atherosclerotic lesions in CRD mice.

## Introduction

Cardiovascular disease, including atherosclerosis, is the leading cause of mortality and morbidity in westernized societies [1–4]. Patients with chronic renal disease (CRD) are more likely to die of cardiovascular disease than renal failure [5]. CRD accelerates the development of atherosclerosis [6–8]. We and others demonstrated that CRD accelerates atherosclerosis and causes excessive vascular inflammation and calcification [9–12].

HMG-CoA (3-hydroxy-3-methylglutaryl-coenzyme A) reductase inhibitors, or statins, are commonly used to lower low-density lipoprotein (LDL) cholesterol levels. Pitavastatin, a new member of statin family, has a unique chemical structure that contributes to multiple pharmacological benefits including potent efficacy for treatment of dyslipidemia, minimal drug-drug interactions, high levels of systemic bioavailability and oral absorption [13, 14]. Cholesterol lowering by statins reduces vascular inflammation and prevents cardiovascular events [15, 16]. Experimental and clinical studies suggest that statins can reduce atherosclerosis through cholesterol-independent effects including improving endothelial function [17, 18], enhancing the stability of atherosclerotic plaques [19, 20], and decreasing vascular inflammation [21, 22]. Clinical evidence suggests that some statins improve kidney function, but whether statin monotherapy reduces atherogenesis in patients with CRD and prevents cardiovascular events in this patient population remain uncertain [5]. We therefore hypothesized that pitavastatin can reduce inflammation in atherosclerotic plaques in CRD.

## Materials and Methods

### Mouse Model of CRD

**Fig 1:**
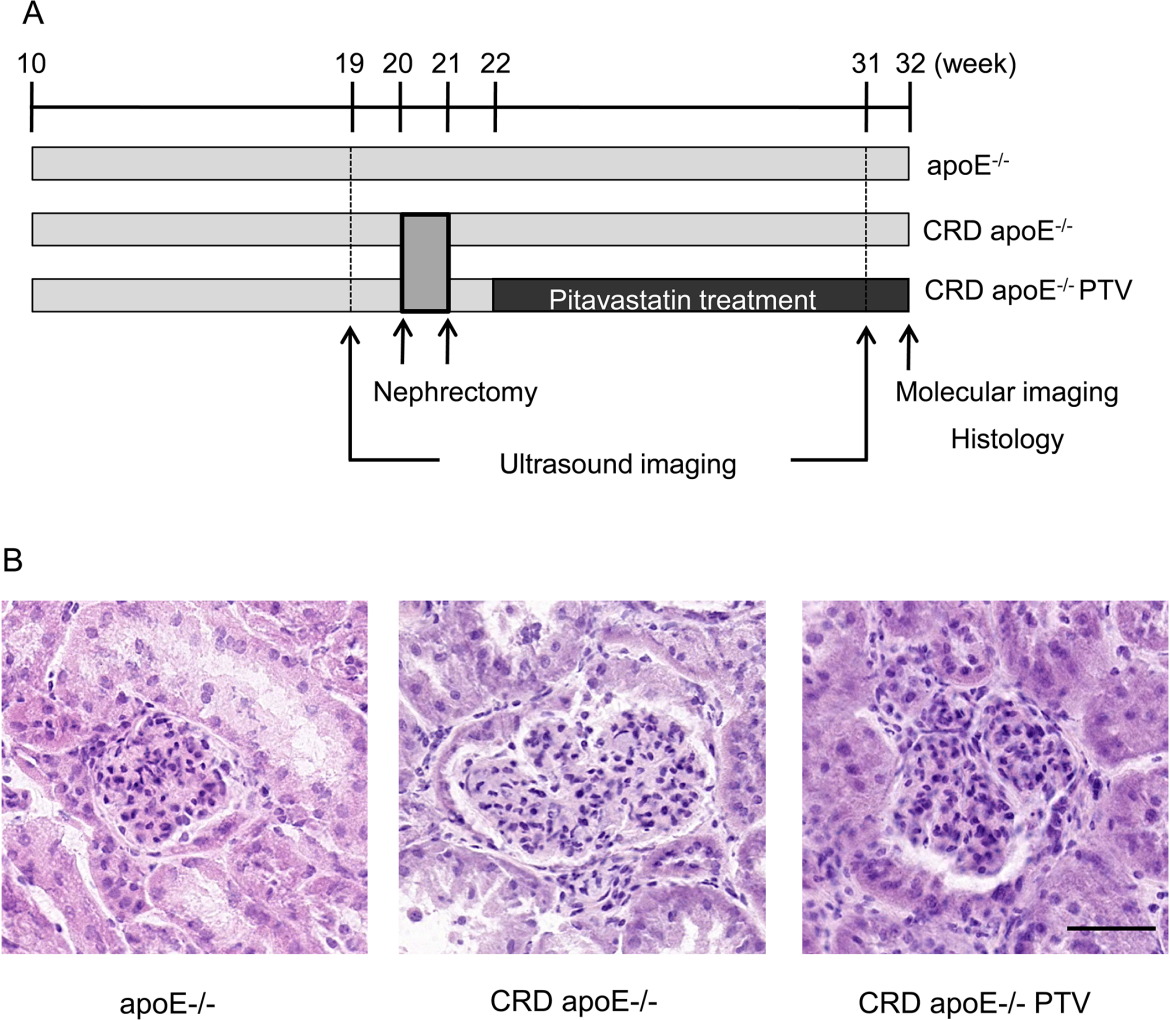
A: Study design. High-cholesterol-fed apoE^-/-^ mice at 19 weeks of age were randomized into control mice (n = 10) and CRD mice treated or untreated with pitavastatin (n = 20 per group). Pitavastatin was administered as a food admixture for 10 weeks starting at 22 weeks. Development of luminal stenosis in brachiocephalic arteries was monitored by ultrasonography at 19 weeks (before nephrectomy) and at 31 weeks. Ex vivo near infrared fluorescence molecular imaging and tissue harvesting for histology were performed at 32 weeks. B: Histological evidence of kidney insufficiency in CRD mice. Hematoxylin and eosin staining demonstrates normal kidney morphology in control apoE^-/-^ mice and enlarged glomeruli in CRD apoE^-/-^ mice treated with or without pitavastatin (Black bar = 50 μm).

Male apolipoprotein E-deficient mice (apoE^-/-^ mice; B6/129 background, 10 weeks old) were purchased from Jackson Laboratory (Bar Harbor, ME, USA). High-fat diet (21% fat and 0.21% cholesterol) was obtained from Research Diets (D12079B, New Brunswick, NJ, USA). All mice were fed an atherogenic diet for a total of 22 weeks and randomized into three groups after 10 weeks of feeding: apoE^-/-^ mice (n = 10), apoE^-/-^ CRD mice (n = 20) and apoE^-/-^ CRD mice treated with pitavastatin (n = 20) (Fig 1A). A two-step procedure was performed to induce chronic renal disease (CRD): left heminephrectomy at 20 weeks of age followed by right total nephrectomy 1 week later [9]. One week after nephrectomy, CRD mice were fed a high-cholesterol diet supplemented with pitavastatin (Kowa Company, Ltd., Tokyo, Japan) at a dose of 100 mg/kg diet (0.01% wt/wt) for 10 weeks (from 22 to 32 weeks of age). To monitor plaque changes we performed an ultrasound echocardiography of aortic arch and brachiocephalic artery at 19 weeks and 31 weeks of age. All mice were euthanized by exsanguination while under deep anesthesia with pentobarbital for ex vivo near-infrared (NIR) fluorescence imaging of brachiocephalic artery and correlative histological analyses at 32 weeks of age. All animal experiments were approved by the Institutional Animal Care and Use Committee of the Animal Research Facility at Beth Israel Deaconess Medical Center (Boston, MA, USA). Animal Protocol: 010–2013 - "Cardiovascular Inflammation and Calcification".

### Blood Biochemistry

Whole blood was drawn from inferior vena cava into heparinized microtubes and centrifuged at 2000g for 10 min at 4°C. Plasma was collected and frozen at -80°C. Plasma levels of total cholesterol, creatinine, urea, phosphate and calcium were measured using commercial kits obtained from BioAssay Systems (Hayward, CA, USA) and BioVision (Milpitas, CA, USA). Plasma Cystatin C was analyzed using ELISA kit from BioVendor (Brno, Czech Republic). Plasma osteopontin (OPN) was detected by ELISA kit purchased from R&D systems (Minneapolis, MN, USA). Pitavastatin concentration in plasma was measured by HPLC method as previously described [23].

### In Vivo Ultrasound Imaging

All mice were anesthetized with 1.5–2% isoflurane in 2% oxygen, and the anterior chest wall was shaved using hair removal solution. An ultrasound biomicroscopy system (Vevo 2100, FUJIFILM VisualSonics, Toronto, Canada) equipped with a 40 MHz mechanical transducer was used to measure the diameter of the brachiocephalic artery. The ascending aorta and brachiocephalic artery were visualized in one plane in the right parasternal long-axis view. The diameter of the brachiocephalic artery was measured at the narrowest point near the origin, perpendicular to the axis of blood flow.

### Macroscopic Fluorescence Reflectance Imaging

Macrophage accumulation in aorta was monitored ex vivo as described previously [24–26]. Cross-linked iron oxide fluorescent nanoparticle (AminoSPARK 750, PerkinElmer, Boston, MA, USA) was intravenously injected via tail vein into the mice 24 hours before imaging. After mice were euthanized, aorta was perfused with saline, dissected and imaged to map the macroscopic NIR fluorescent signals elaborated by AminoSPARK 750 (excitation/emission: 750/770 nm) in a fluorescent reflectance system (Image Station 4000MM, Eastman Kodak Co., New Haven, CT, USA). The sum of the fluorescence intensity in brachiocephalic arteries was subtracted to the background level.

### Histopathological Assessment and Morphological Characterization of Atherosclerotic Plaques and Kidney

Tissue samples were snap-frozen in Frozen Section Compound (VWR International, West Chester, PA, USA) and 6-μm serial sections were cut and stained with hematoxylin and eosin for overall morphology. Images were captured with a digital camera (DS-Fi1c, Nikon, Melville, NY, USA). To assess luminal stenosis, whole lumen diameters and residual lumen diameters were measured at the origin of the brachiocephalic artery, perpendicular to the axis of blood flow, using NIS-Elements AR 3.10 (Nikon Instruments, Melville, NY, USA). Residual lumen was shown as a percentage of the residual lumen diameter compared with the whole lumen diameter. The sections were stained for the presence of calcium phosphate crystals using von Kossa method. The black silver staining indicative of calcium phosphate deposition was quantified within atherosclerotic plaques in brachiocephalic arteries using computer assisted imaging analysis [24]. Immunohistochemistry for macrophages (rat monoclonal antibody against mouse Mac3, BD Biosciences, San Jose, CA, USA) and osteopontin (goat polyclonal antibody, ab11503, Abcam, Cambridge, MA, USA) was performed using avidin-biotin peroxidase method. The reaction was visualized with a 3-amino-9-ethylcarbazole substrate (AEC, Sigma-Aldrich, St Louis, MO, USA). The positive area of red reaction product associated with macrophage accumulation or osteopontin expression was quantified within atherosclerotic lesions in brachiocephalic arteries using computer assisted imaging analysis [24].

### In Vitro Calcium Deposition in Vascular Smooth Muscle Cells

Mouse smooth muscle cells were cultured in DMEM containing 10% fetal bovine serum (FBS), 3 mM calcium and 2 mM phosphate with or without 50 nM pitavastatin (PTV). After 7 days, cells were decalcified with 0.6 M HCl for 24 hours. The calcium content in HCl supernatant was measured colorimetrically by the *o*-cresolphthalein complexone method (Calcium Colorimetric Assay Kit, BioVision). The cells were then washed once with PBS and solubilized with 0.1 M NaOH and 0.1% SDS. The protein amount was determined using a BCA protein assay kit (Thermo Scientific, Rockford, IL, USA). The calcium content was normalized to cellular protein content.

### Osteopontin Expression in Murine Peritoneal Macrophages

Peritoneal macrophages were prepared as described before [27]. Macrophages were cultured in RPMI 1640 medium supplemented with 10% FBS and antibiotics (penicillin, streptomycin and amphotericin B). Cells were preincubated with either DMSO control or pitavastatin at dose of 100 nM or 300 nM for 9 hours, followed by stimulation with either 5 mM phosphate or calcium/phosphate (3 mM calcium and 2 mM phosphate) for another 12 hours. Total RNA samples were extracted using an Illustra RNAspin Mini kit (GE Healthcare, Piscataway, NJ, USA) and cDNAs were synthesized using a high capacity cDNA reverse transcription kit (Applied Biosystems, Carlsbad, CA, USA). Real-time PCR was performed using Taqman probes for osteopontin and GAPDH on a 7900HT fast real-time PCR system (Applied Biosystems). Relative expression of osteopontin was normalized by GAPDH.

### Statistical Analysis

Statistical significance between multiple groups was analyzed by One-way ANOVA followed by the Tukey post hoc test using GraphPad Prism 5 (San Diego, CA, USA). Data are presented as mean ± SEM. *P* values less than 0.05 were considered significant.

## Results

### Pitavastatin Does Not Improve Impaired Renal Function in Nephrectomized Mice

**Fig 2:**
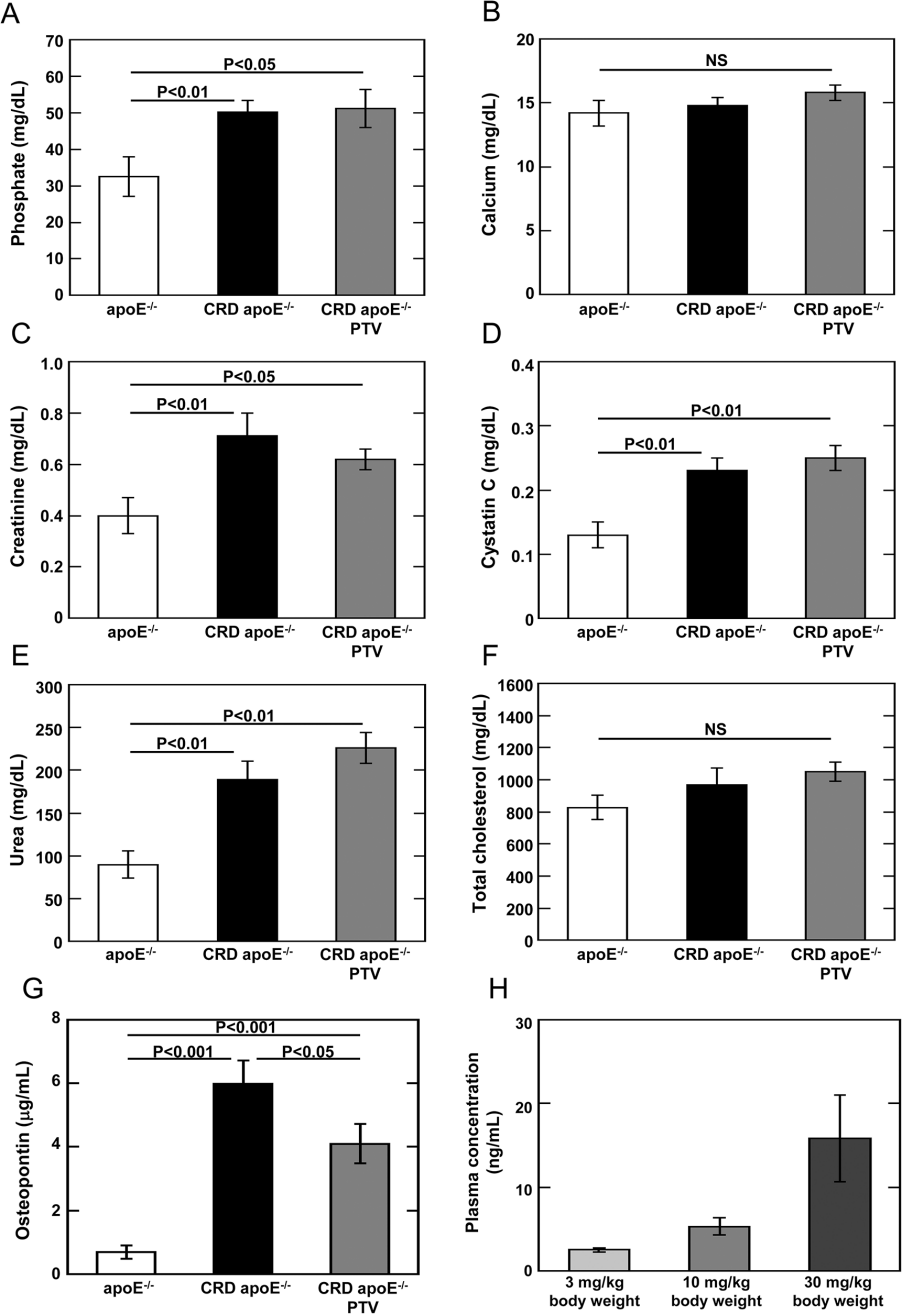
Blood biochemistry and plasma levels of osteopontin and pitavastatin. Mouse plasma was prepared from 32-weeks old apoE^-/-^ mice fed with high-fat diet. Levels of phosphate (A), calcium (B), creatinine (C), cystatin C (D), urea (E), total cholesterol (F) and osteopontin (G) were measured in plasma from apoE^-/-^ mice (n = 10), CRD apoE^-/-^ mice (n = 14) and CRD apoE^-/-^ mice treated with pitavastatin (CRD apoE^-/-^ PTV, n = 18). Data are shown as mean ± SEM. H: Plasma concentration of pitavastatin given as food admixture in mice. ApoE^-/-^ mice were fed a chow supplemented with pitavastatin at doses of 30, 100 and 300 mg/kg diet (0.003, 0.01 and 0.03% wt/wt) for 2 weeks. These doses were equivalent to 3, 10 and 30 mg pitavastatin/kg body weight, respectively. Mice treated with pitavastatin at a dose of 100 mg/kg diet had plasma concentration of 5.3 ± 1.0 ng/mL. Data are shown as mean ± SEM (n = 5).

Blood biochemistry was conducted using plasma samples from 32-week-old apoE^-/-^ control mice or apoE^-/-^ CRD mice. Mice fed with 10 milligram pitavastatin per kilogram diet had the blood concentration of 5.3 ± 1.0 ng/mL, which was equivalent to the concentration in human (Fig 2H). Plasma levels of phosphate (P<0.01), creatinine (P<0.05), cystatin C (P<0.01) and urea (P<0.01) were significantly higher in CRD apoE^-/-^ mice than apoE^-/-^ controls (Fig 2A, 2C, 2D and 2E). These results validate the establishment of the severe CRD model. Pitavastatin treatment did not improve parameters associated with this CRD condition likely due to irreversability of post-nephrectomy kidney function (Fig 2A, 2C, 2D and 2E). Kidney morphology assessed by hematoxylin and eosin staining demonstrated no significant difference between apoE^-/-^ CRD mice and those treated with pitavastatin (Fig 1B). In addition, pitavastatin had no significant impact on the levels of total cholesterol (Fig 2F) and triglycerides (S1B Fig), which was consistent with previous studies in murine models of atherosclerosis [28–30]. At 32 weeks, pitavastatin treatment did not affect body weight of apoE^-/-^ CRD mice (S1A Fig).

### Pitavastatin Decreases Pro-inflammatory osteopontin in Plasma of CRD mice

We determined the expression levels of osteopontin in plasma samples. CRD apoE^-/-^ mice had higher plasma osteopontin levels compared to control apoE^-/-^ mice (P<0.001). Pitavastatin treatment reduced plasma osteopontin levels (P<0.05, Fig 2G).

### Pitavastatin Reduces Stenosis in Brachiocephalic Arteries

**Fig 3:**
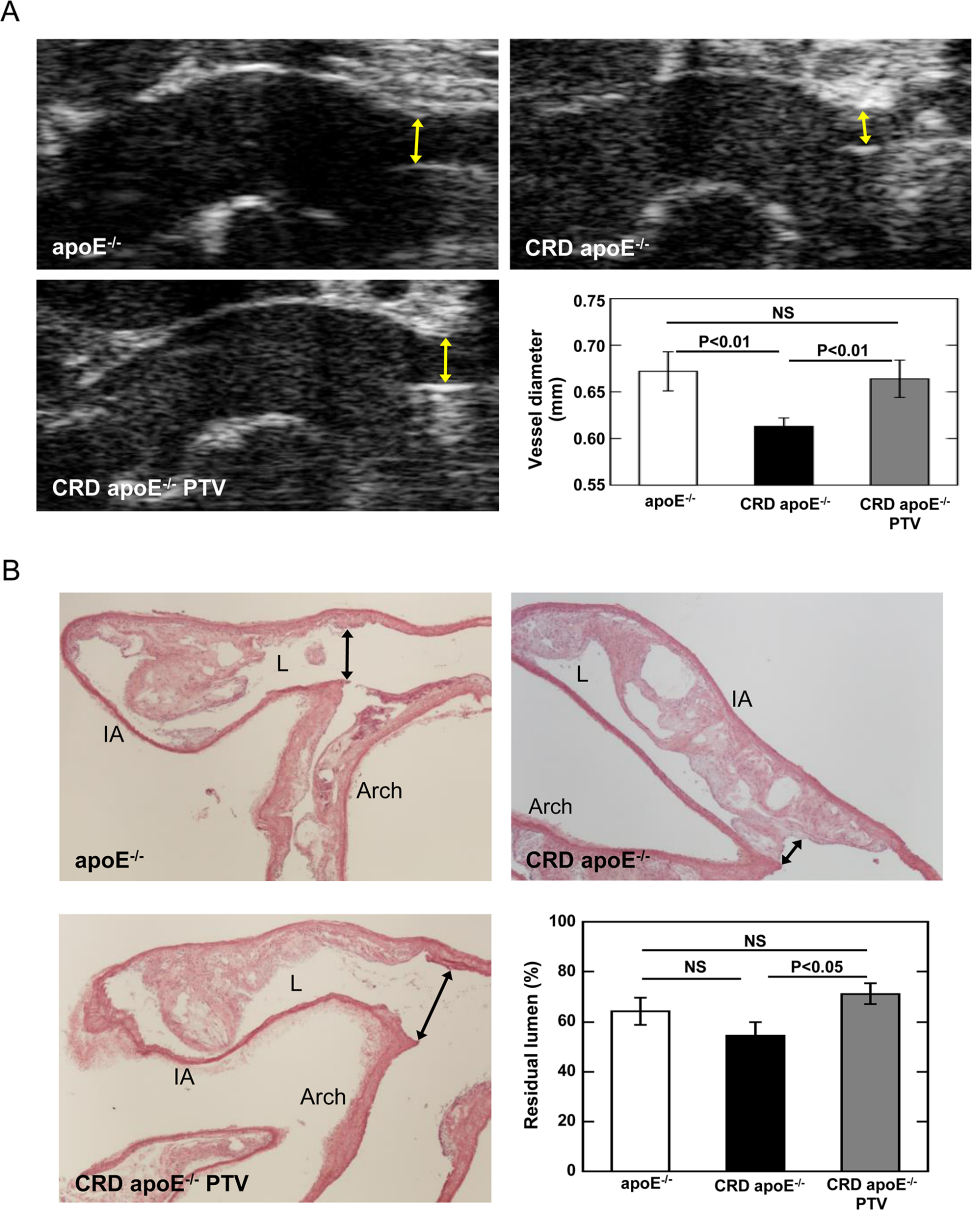
A: In vivo ultrasound imaging of brachiocephalic artery. Luminal vessel diameter of brachiocephalic artery was monitored by ultrasonography at 19 weeks and at 31 weeks. Representative ultrasound imaging of control apoE^-/-^ mice (n = 6), CRD apoE^-/-^ mice (n = 12), and CRD apoE^-/-^ mice treated with pitavastatin (CRD apoE^-/-^ PTV, n = 13) at 31 weeks. Yellow arrows indicate luminal diameters. Quantitative assessment of luminal diameters showed that pitavastatin treatment significantly reduced stenosis in CRD mice at 31 weeks. B: Histological analysis of brachiocephalic arteries. Hematoxylin and eosin staining was performed on sections of brachiocephalic arteries from control apoE^-/-^ mice (n = 7), CRD apoE^-/-^ mice (n = 12), and CRD apoE^-/-^ mice treated with pitavastatin (CRD apoE^-/-^ PTV, n = 16). Representative images were shown (Arch: aortic arch; IA: brachiocephalic artery; L: lumen). Black arrows indicate residual lumen diameters. Quantitative assessment of residual lumen (%) at the origin of brachiocephalic arteries was shown as mean ± SEM.

Development of luminal stenosis in brachiocephalic artery was monitored by ultrasonography in vivo at 19 weeks (before nephrectomy) and at 31 weeks (one week before sacrifice). Luminal diameter did not differ in all three groups of mice at 19 weeks (Data not shown). Luminal diameter of the brachiocephalic arteries in CRD apoE^-/-^ mice at 31 weeks of age decreased 14% (P<0.001) compared with that at 19 weeks, which indicates a progressive development of stenosis. At 31 weeks, CRD apoE^-/-^ mice decreased vessel diameters as compared to apoE^-/-^ controls (0.67 ± 0.02 mm vs. 0.61 ± 0.01 mm; P<0.01, Fig 3A), while pitavastatin treatment increased the vessel diameter in CRD apoE^-/-^ mice (0.61 ± 0.01 mm vs. 0.66 ± 0.02 mm; P<0.01, Fig 3A). Similar results were observed by histological analysis (Fig 3B). Residual lumen at the origin of the brachiocephalic artery was measured to evaluate luminal stenosis. CRD apoE^-/-^ mice tended to have smaller residual lumens than control apoE^-/-^ mice (P = 0.23, Fig 3B). Pitavastatin treatment increased residual lumens in brachiocephalic arteries of CRD apoE^-/-^ mice (54.5 ± 5.2% vs. 71.2 ± 4.2%; P<0.05, Fig 3B).

### Pitavastatin Reduces Vascular Inflammation in Brachiocephalic Arteries

**Fig 4:**
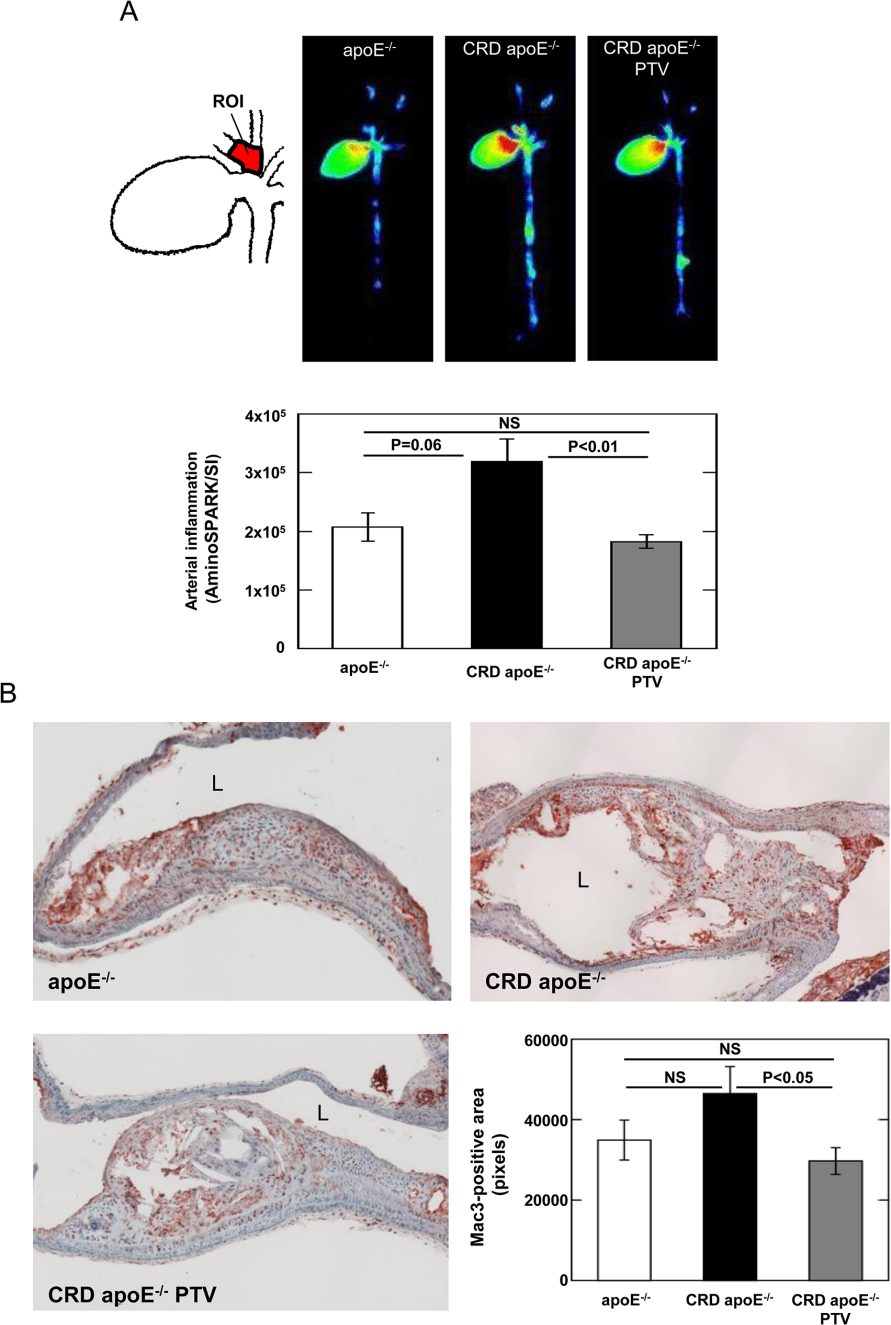
Pitavastatin reduces macrophage accumulation in brachiocephalic arteries of CRD mice. A: Ex vivo fluorescence reflectance imaging (FRI) analysis. Representative images of the fluorescence intensity in the entire aorta were shown as red-green-blue (RGB) readout. Quantitative assessment of the signal intensity in the brachiocephalic artery (ROI) was shown as mean ± SEM. B: Mac3 immunostaining of brachiocephalic arteries. Representative images of macrophage accumulation within atherosclerotic lesions in brachiocephalic arteries of control apoE^-/-^ mice (n = 9), CRD apoE^-/-^ mice (n = 9), and CRD apoE^-/-^ mice treated with pitavastatin (CRD apoE^-/-^ PTV, n = 15). L indicates lumen. Quantitative assessment of Mac3-postive area was shown as mean ± SEM.

Macroscopic fluorescence reflectance imaging (FRI) was conducted to detect macrophage accumulation in the aorta and brachiocephalic arteries 11 weeks after the nephrectomy. The fluorescence intensity in brachiocephalic arteries (ROI shown in Fig 4A) in CRD apoE^-/-^ mice had a trend to increase compared to that in apoE^-/-^ control mice (P = 0.06, Fig 4A). Pitavastatin treatment reduced the fluorescence intensity in brachiocephalic arteries of CRD apoE^-/-^ mice (-42.8 ± 3.5%, P<0.01, Fig 4B), suggesting decreased macrophage accumulation. Immunostaining of Mac3 on tissue sections of brachiocephalic arteries concurred these results. Macrophage-positive area in brachiocephalic arteries decreased in CRD apoE^-/-^ mice treated with pitavastatin compared with untreated CRD apoE^-/-^ mice (-36.3 ± 7.1%; P<0.05, Fig 4B).

### Pitavastatin Decreases Osteopontin Expression within Atherosclerotic Lesions in Brachiocephalic Arteries of CRD ApoE^-/-^ mice

**Fig 5:**
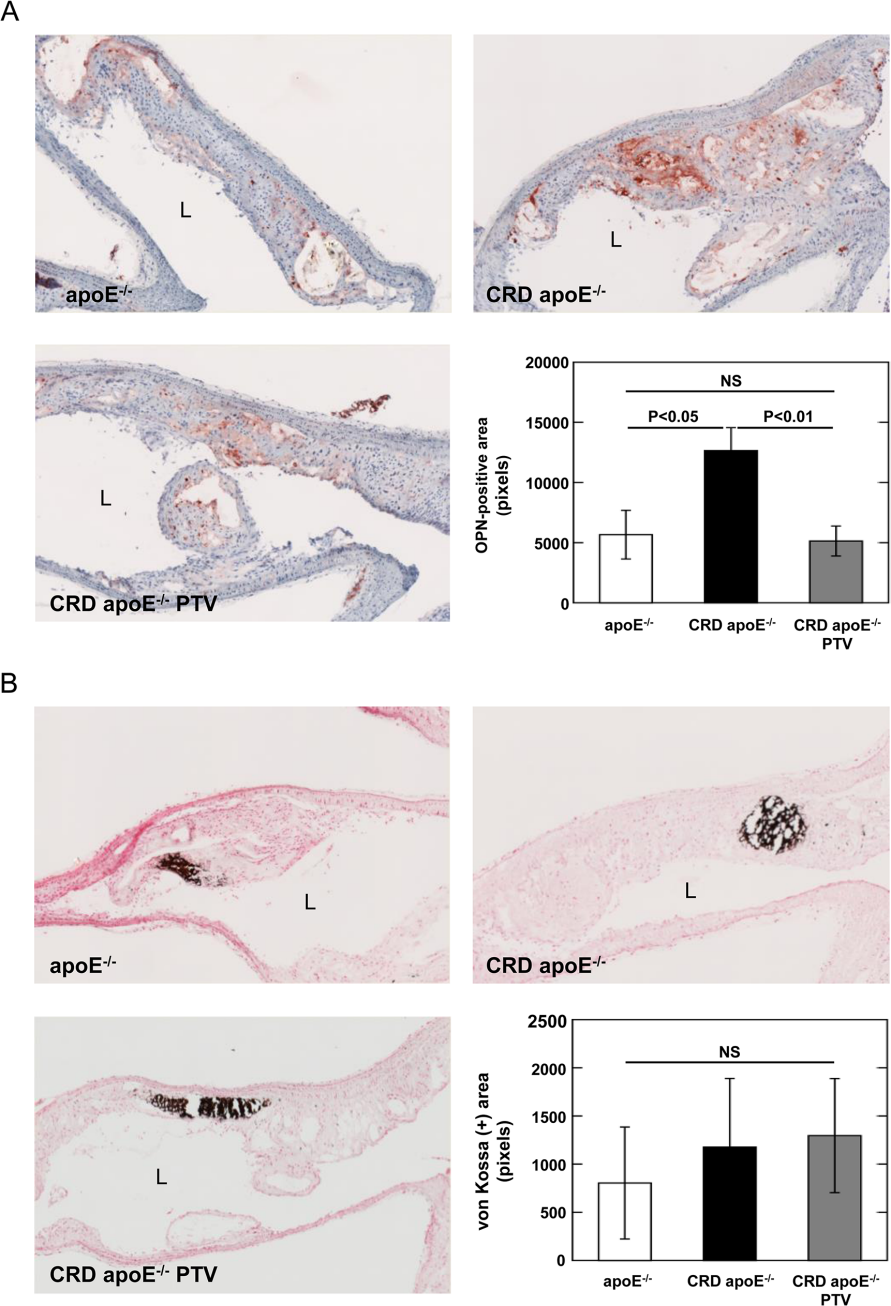
A: Pitavastatin reduces osteopontin expression in brachiocephalic arteries of CRD mice. Representative images of osteopontin immunostaining within atherosclerotic plaques in brachiocephalic arteries of control apoE^-/-^ mice (n = 8), CRD apoE^-/-^ mice (n = 12), and CRD apoE^-/-^ mice treated with pitavastatin (CRD apoE^-/-^ PTV, n = 14). L indicates lumen. Quantitative assessment of OPN-positive area was shown as mean ± SEM. B: Pitavastatin has no significant effect on calcification in brachiocephalic arteries of CRD mice. Representative images of advanced calcification within atherosclerotic lesions in brachiocephalic arteries of control apoE^-/-^ mice (n = 6), CRD apoE^-/-^ mice (n = 11), and CRD apoE^-/-^ mice treated with pitavastatin (CRD apoE^-/-^ PTV, n = 15). Quantitative assessment of von Kossa-positive area was shown as mean ± SEM. L indicates lumen.

Since pitavastatin treatment reduced osteopontin levels in plasma of CRD apoE^-/-^ mice (Fig 2G), we further determined the protein levels of osteopontin in brachiocephalic arteries by immunohistochemical analysis (Fig 5A). The brachiocephalic arteries of CRD apoE^-/-^ mice had greater osteopontin expression than control apoE^-/-^ mice (P<0.05). Ten-week pitavastatin treatment remarkably decreased the osteopontin protein expression in plaques compared with untreated CRD apoE^-/-^ mice (-59.4 ± 9.8%; P<0.01, Fig 5A).

### Pitavastatin Does Not Reduce Calcification in Atherosclerotic Plaques in Brachiocephalic Arteries and Cultured Vascular Smooth Muscle Cells

**Fig 6:**
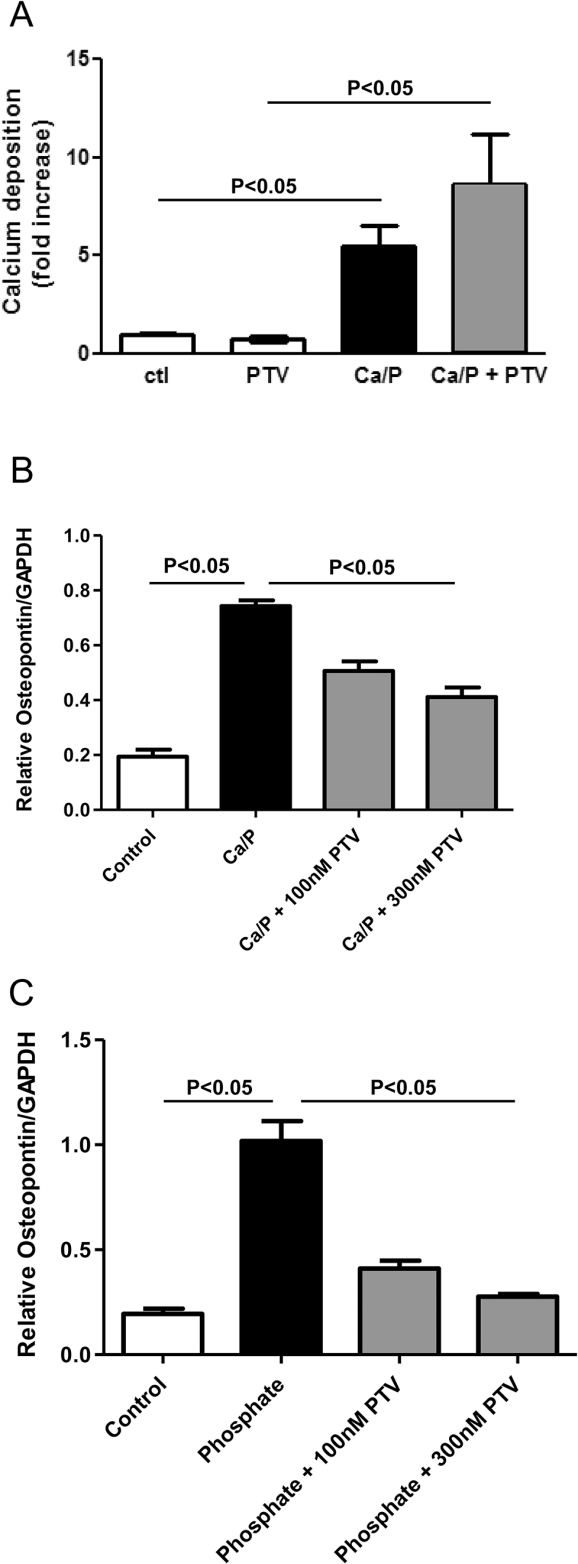
A: Pitavastatin has no significant effect on calcification in vascular smooth muscle cells. Mouse vascular smooth muscle cells were treated with or without 50 nM pitavastatin (PTV) in the presence of calcium/phosphate (Ca/P, 3 mM calcium and 2 mM phosphate) for 7 days. Calcium deposition was determined by o-cresolphthalein complexone method and normalized by cellular protein content. Data are shown as mean ± SEM (n = 3 each group). B and C: Pitavastatin reduces osteopontin mRNA expression in peritoneal macrophages. Macrophages were preincubated with either DMSO control or pitavastatin (100 nM or 300 nM) and followed by stimulation with calcium/phosphate (Ca/P, 3 mM calcium and 2 mM phosphate or 5 mM phosphate). mRNA levels of osteopontin (B,C) were determined by real-time PCR and normalized by mRNA levels of GAPDH. Data are shown as mean ± SEM (n = 6 each group).

Histological analysis demonstrated that von Kossa-positive area associated with advanced calcification in brachiocephalic arteries did not differ in CRD apoE^-/-^ and control apoE^-/-^ mice (Fig 5B). Quantitative measurement of the levels of calcium and phosphate confirms that pitavastatin did not alter the deposition of calcium and phosphate in brachiocephalic arteries (S2A and S2B Fig), even though a significant correlation was observed between the levels of calcium and phosphate (S2C Fig). All these data suggest that pitavastatin did not significantly affect calcium deposition in brachiocephalic arteries. We then evaluated the effects of pitavastatin on calcification in mouse vascular smooth muscle cells (VSMCs) in vitro. We found a 5.5-fold increase of calcium deposition in VSMCs treated with 3 mM calcium and 2 mM phosphate as compared to control VSMCs (Fig 6A). Pitavastatin treatment did not affect calcium deposition in VSMCs induced by calcium/phosphate (Fig 6A).

### Pitavastatin Significantly Inhibits the Expression of Osteopontin in Mouse Peritoneal Macrophages

We found that pitavastatin treatment reduced osteopontin levels in plasma and brachiocephalic arteries in CRD apoE^-/-^ mice. To further explore the molecular mechanisms, we tested the effect of pitavastatin on mouse peritoneal macrophages stimulated with an elevated concentration of calcium/phosphate or phosphate. Calcium/phosphate (3 mM calcium/2 mM phosphate or 5 mM phosphate) increased osteopontin mRNA levels, which were attenuated by 300 nM pitavastatin treatment (Fig 6B and 6C).

## Discussion

The major findings of this study i) showed that the induction of CRD by 5/6 nephrectomy promotes vascular inflammation within atherosclerotic lesions in brachiocephalic arteries of apoE^-/-^ mice; ii) demonstrated the significant reduction of stenosis in brachiocephalic arteries of CRD apoE^-/-^ mice treated by pitavastatin with no substantial changes in cholesterol levels; iii) proved that macrophage accumulation in brachiocephalic arteries decreased by pitavastatin treatment for 10 weeks; iv) demonstrated that pitavastatin treatment reduces pro-inflammatory osteopontin in vivo and in cultured primary macrophages; and v) revealed that pitavastatin does not affect vascular calcification in the animal model of CRD, induced by 5/6 nephrectomy. This study therefore demonstrates that a clinically-achievable concentration of pitavastatin reduces arterial inflammation within atherosclerotic plaques in the CRD mouse model through the mechanism independent of its lipid lowering effects.

Pitavastatin treatment significantly reduced pro-inflammatory and pro-osteogenic osteopontin levels in plasma and brachiocephalic arteries in CRD apoE^-/-^ mice. Multifunctional osteopontin is biosynthesized by many cell types, including macrophages and VSMCs. The expression of osteopontin can be stimulated by various pro-inflammatory cytokines [31–33], endotoxin [34] and extracellular inorganic phosphate [35]. Here we first show that pitavastatin treatment significantly attenuates the expression of osteopontin induced by phosphate/calcium or phosphate alone in primary peritoneal macrophages. Our finding is consistent with previous report that pitavastatin (NK-104) treatment reduces osteopontin expression in cultured rat aortic SMCs and aortic tissue from diabetic rats [36]. Further, a recent study reported that simvastatin reduces plasma levels of osteopontin in patients with CAD [37]. These sets of data indicate that abnormally high levels of phosphate (5 mM) in the plasma of CRD apoE^-/-^ mice may induce osteopontin expression in macrophages and VSMCs, which can be attenuated by pitavastatin treatment. However, the clinical relevance of the inhibitory effect of pitavastatin in phosphate-induced osteopontin expression warrants further investigation.

Earlier studies have demonstrated that both NF-κB and activator protein-1 (AP-1) pathways play important roles in the transcriptional expression of osteopontin induced by cytokines and LPS [33, 34]. We found no difference of osteopontin mRNA expression between classically activated M1 macrophages induced by IFN-γ and alternative M2 macrophages stimulated by IL-4 (S3 Fig). Two functionally distinct NF-κB activation inhibitors (Bay 11–7802 and JSH-23) have no significant influence of osteopontin mRNA induced by phosphate (S4 Fig). It was previously reported that osteopontin gene promoter contains glucocorticoid response element and glucocorticoid receptor signaling regulates osteopontin transcription induced by phosphate in murine cementoblasts [38]. Therefore, pitavastatin may suppress phosphate-mediated osteopontin transcription by modulating glucocorticoid receptor signaling.

The beneficial effects of pitavastatin on increased vascular inflammation in CRD mice may be explained at least partially by reduction of arterial osteopontin expression. High levels of osteopontin protein was detected in macrophages within atherosclerotic plaques [39]. Pitavastatin treatment could attenuate osteopontin expression within arterial lesions (Fig 5A) through either lowering macrophage accumulation (Fig 4B) or directly suppressing osteopontin expression on macrophages (Fig 6B and 6C). Osteopontin has numerous functions, including inhibition of calcium deposition in early stages, promotion of calcification in advanced plaques, and induction of atherosclerotic inflammation [40–43]. In a previous study, neutralizing antibodies directed against osteopontin inhibited rat carotid neointimal thickening after endothelial denudation [40]. The results from osteopontin and apoE double-deficient mice showed that osteopontin promotes atherogenesis [41]. More importantly, Shao et al. demonstrated that osteopontin has multifunctional and stage-specific roles in atherosclerosis in male LDL receptor-deficient mice [42]. Full-length phosphorylated osteopontin is relatively protease resistant and is an inhibitor of calcification [42]. On the other hand, the N-terminal fragment of osteopontin that is processed by thrombin-mediated proteolysis in chronic inflammatory diseases including atherosclerosis, promotes vascular inflammation and calcification [42]. Taken together, these results suggested that osteopontin plays an important role in atherosclerosis. Thus, pitavastatin treatment could inhibit vascular inflammation through the reduction of arterial osteopontin expression. Whether osteopontin plays a causal role in the beneficial effect of pitavastatin on vascular inflammation and luminal stenosis in CRD deserves further investigations.

In our study, pitavastatin did not improve the impaired renal function by nephrectomy. Pitavastatin treatment did not improve plasma levels of phosphate, creatinine, cystatin C, and urea in CRD apoE-/- mice. Among these, phosphate is the most important parameter in blood, which was increased in our model up to 5 mM corresponding to 3–4 stage of CRD (~50 mg/dL). Clinical reports further suggest that elevated serum phosphate concentrations are associated with a substantially greater risk of end-stage CRD, and that risk increased up to 5-fold for each 1 mg/dL increment in the mean serum phosphate concentration [44]. Furthermore, the recent reports noted that hyperphosphatemia plays a pivotal role in promoting vascular calcification by modifying Klotho-FGF23 axis [45, 46]. In our study, pitavastatin did not attenuate in vivo calcification in the presence of elevated levels of phosphate, suggesting that the inhibition of arterial inflammation may not be enough to reduce arterial calcification in late-stage CRD. However, it is plausible that pitavastatin may have the beneficial effect on vascular calcification in early-stage CRD as supported by the study by Ivanovski et al. [47]. In their model apoE-/- mice were fed with normal chow diet and CRD was induced by left total nephrectomy. The authors demonstrated that simvastatin treatment reduced vascular calcification in the less severe renal dysfunction characterized by lower levels of urea and no changes in serum phosphate.

Previous preclinical evidence demonstrated that some statins, including pitavastatin, improves renal function [48, 49]. However, in our mechanical nephrectomy model with irreversible kidney function corresponding to the CRD stage 4 from the parameters including urine creatinine, plasma creatinine, and body surface area estimated from body weight, pitavastatin did not change the renal function. Our findings in a mouse model of late-stage CRD was consistent with the latest clinical study (SHARP), which reported that lowering LDL cholesterol with combined simvastatin and ezetimibe therapy has no significant effect on the progression of kidney disease in patients with late stage CRD [50]. Nevertheless, pitavastatin reduced vascular inflammation. Our study did not attempt to examine whether pitavastatin attenuates renal function, but to test the specific biological hypothesis that pitavastatin can reduce vascular inflammation in mice induced by CRD. The present study provides the evidence of a preventive role of pitavastatin in vascular inflammation in CRD through the mechanism independent of its lipid lowering effects. But the study did not establish that anti-inflammatory effects of pitavastatin also reduce arterial calcification in late-stage CRD. These results may indicate that statin administration in the earlier stages of CRD may be required to prevent calcification, and may also suggest that, in addition to anti-inflammatory treatment, therapies focusing more specifically on the processes of ectopic mineralization can retard or regress arterial calcification. This area deserves further preclinical and clinical investigations.

## Supporting Information

S1 Fig Mouse body weight and plasma levels of triglycerides. (TIF)

S2 Fig Pitavastatin has no significant effect on the deposition of calcium and phosphate in brachiocephalic arteries. (TIF)

S3 Fig Osteopontin mRNA expression in M1 and M2 macrophages. (TIF)

S4 Fig NF-κB activation inhibitors have no effect on the osteopontin mRNA expression induced by phosphate in peritoneal macrophages. (TIF)

S1 File Mouse body weight and plasma levels of triglycerides. (DOCX)

S2 File Quantification of Calcium and Phosphate in Brachiocephalic Arteries. (DOCX)

S3 File Osteopontin Expression in Polarized M1 or M2 Macrophages. (DOCX)

S4 File Effect of NF-κB Activation Inhibitors on the Osteopontin Expression in Peritoneal Macrophages. (DOCX)
