# Supplementary figures and images for: Pitavastatin Reduces Inflammation in Atherosclerotic Plaques in Apolipoprotein E-Deficient Mice with Late Stage Renal Disease
Source: PLoS One. 2015 Sep 14;10(9):e0138047. doi: 10.1371/journal.pone.0138047 (PMC4569429; doi:10.1371/journal.pone.0138047)

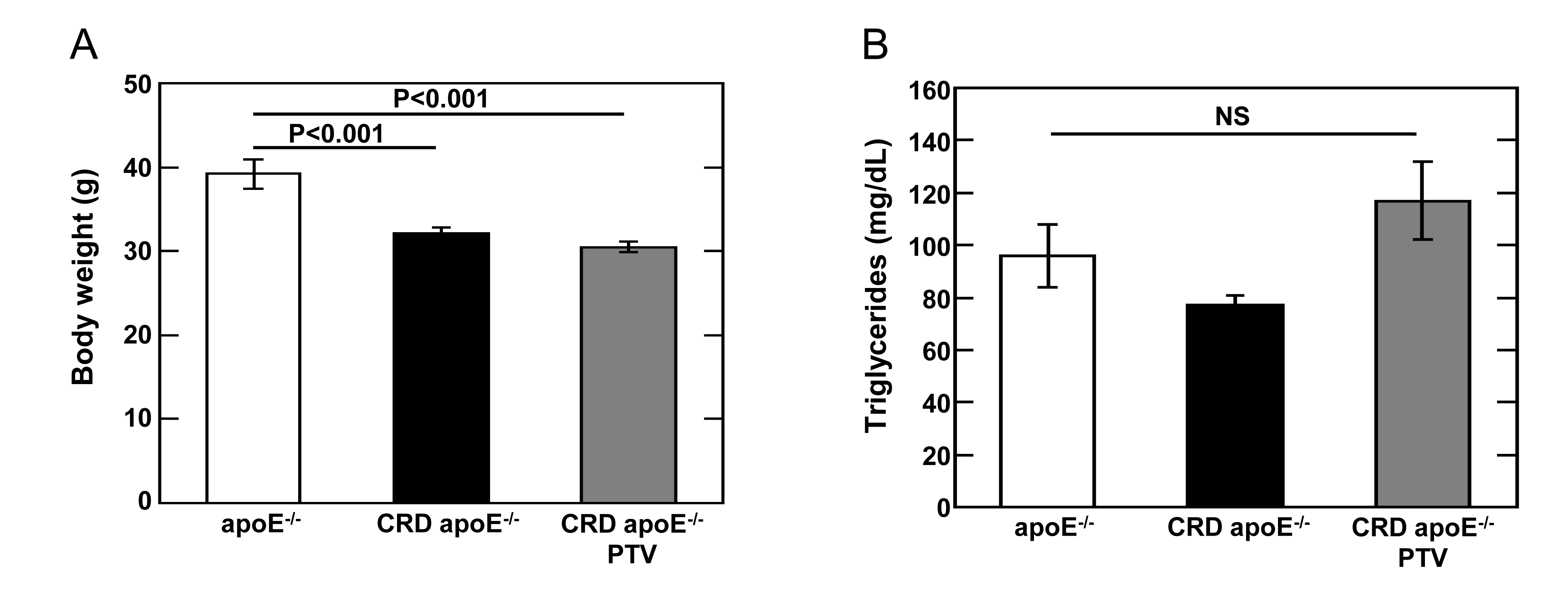

Supplement: S1 Fig — (TIF) [file pone.0138047.s001.tif]

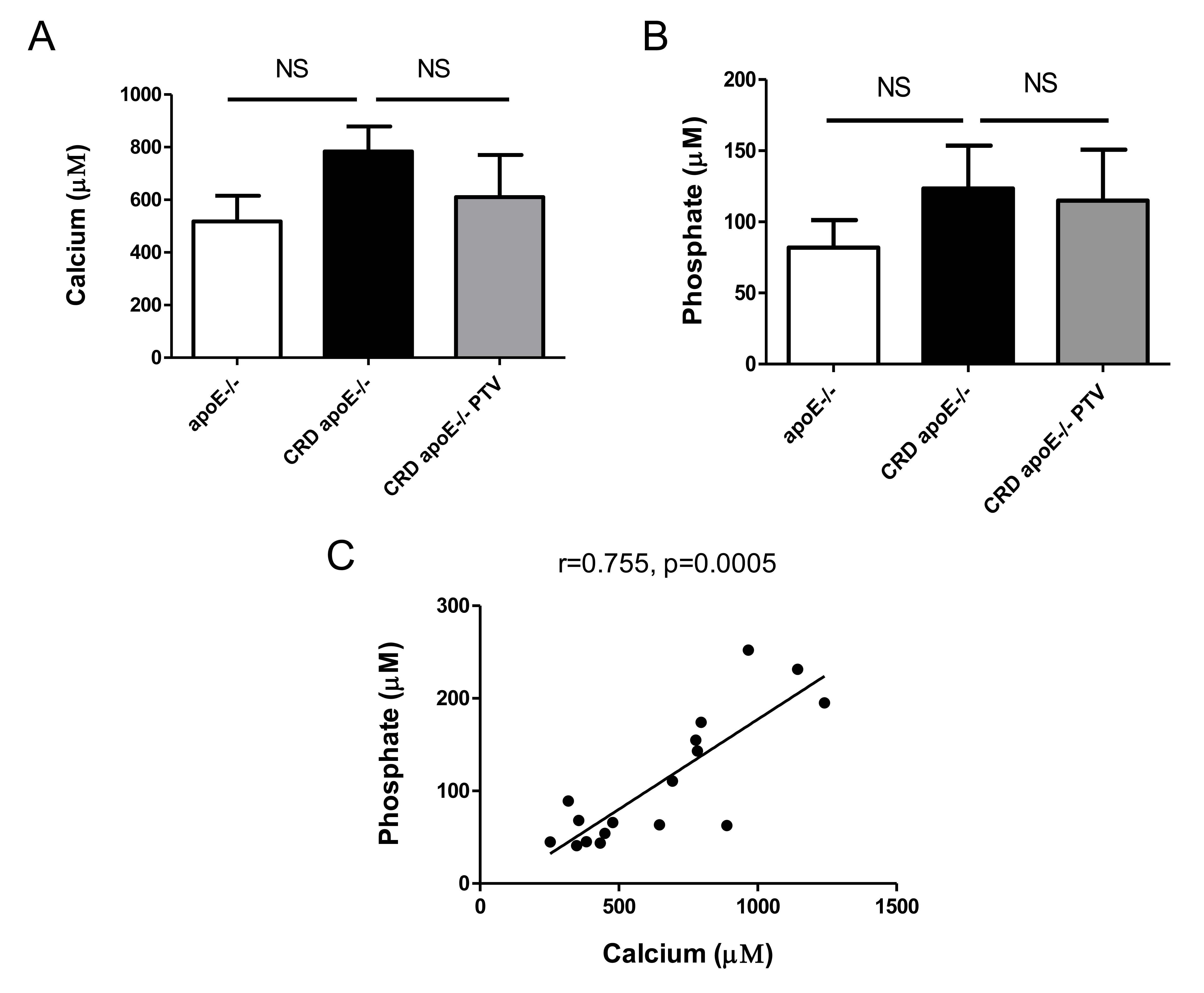

Supplement: S2 Fig — (TIF) [file pone.0138047.s002.tif]

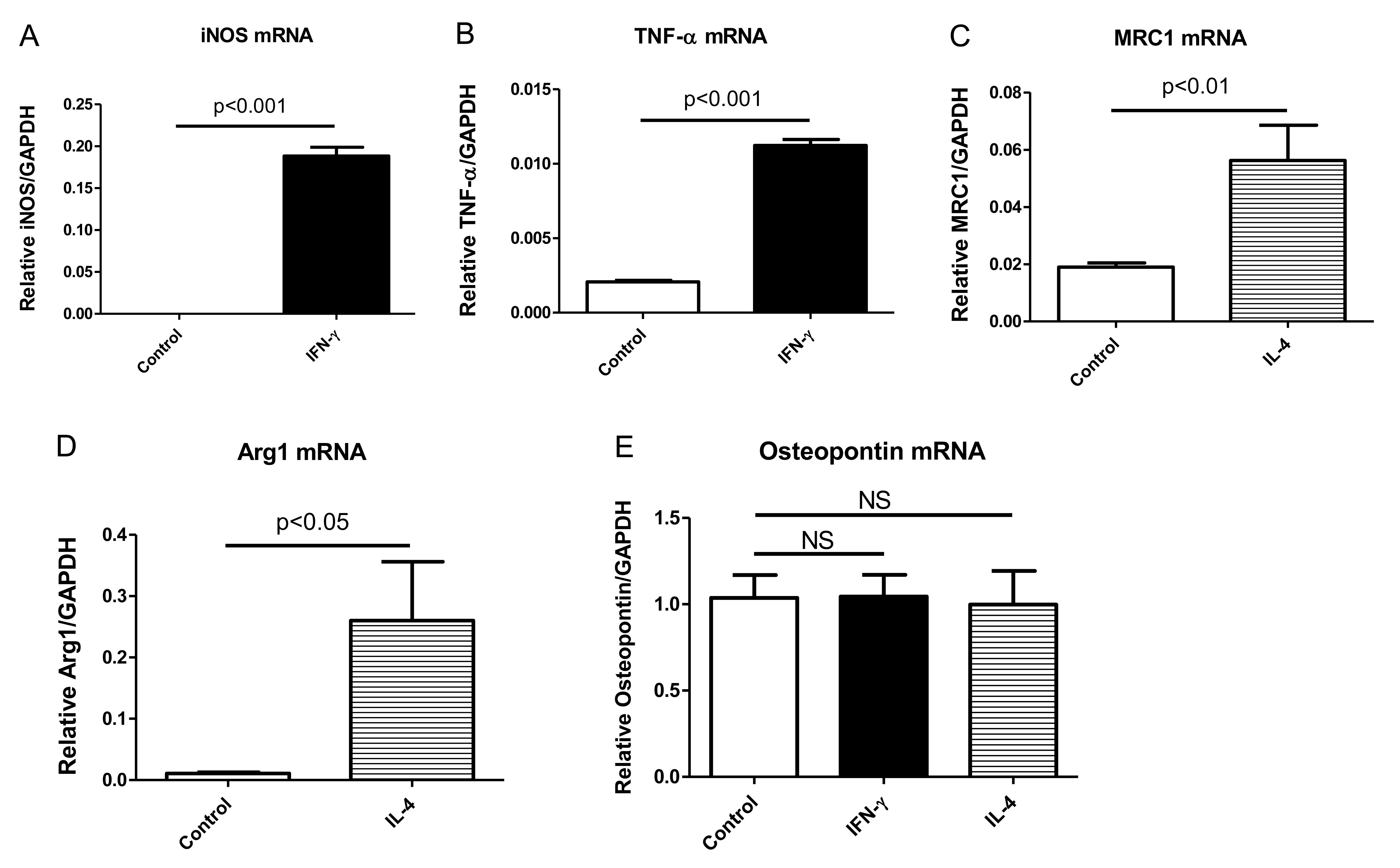

Supplement: S3 Fig — (TIF) [file pone.0138047.s003.tif]

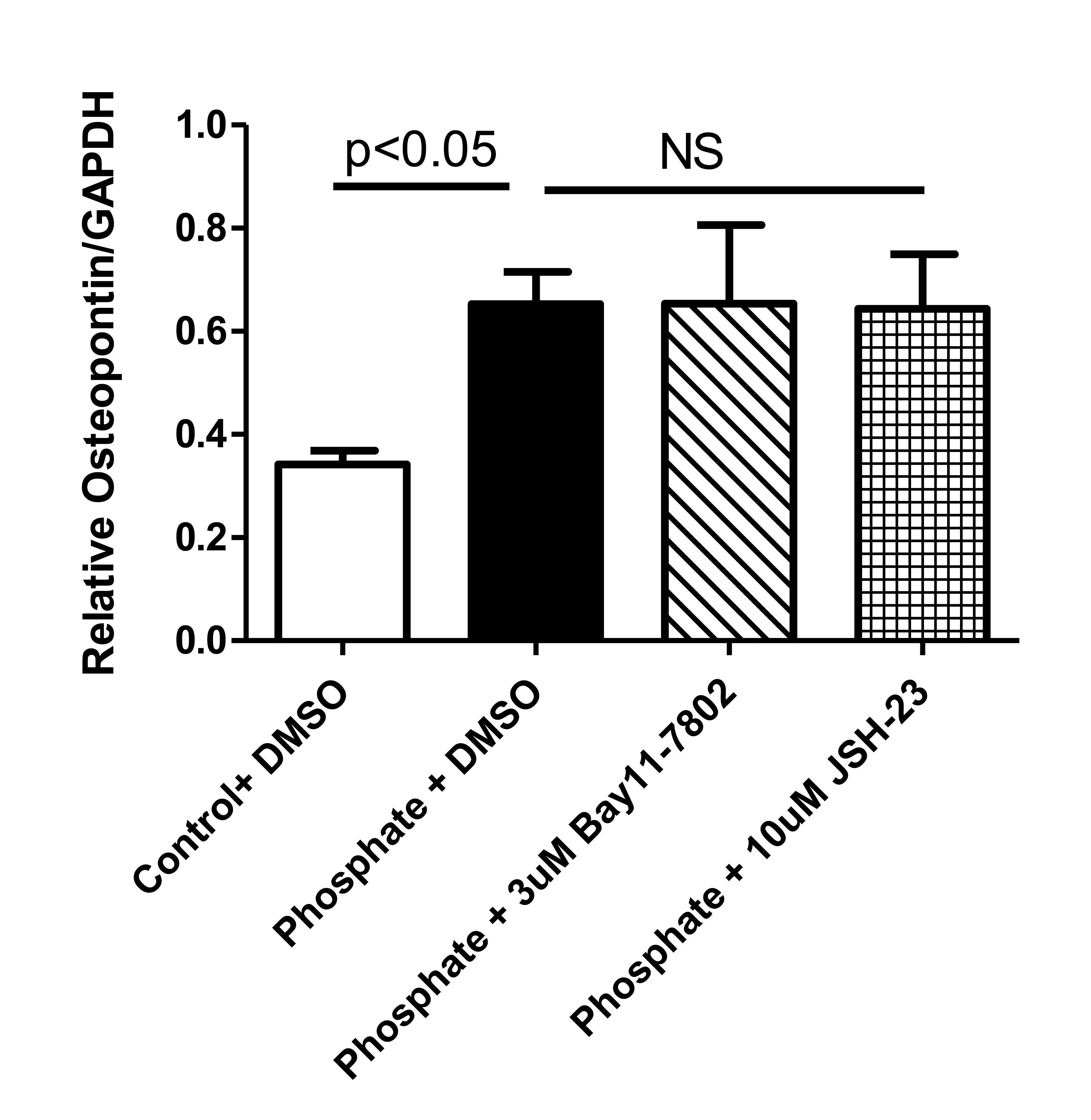

Supplement: S4 Fig — (TIF) [file pone.0138047.s004.tif]
