# Supplementary material for: Pitavastatin Reduces Inflammation in Atherosclerotic Plaques in Apolipoprotein E-Deficient Mice with Late Stage Renal Disease
Source: PLoS One. 2015 Sep 14;10(9):e0138047. doi: 10.1371/journal.pone.0138047 (PMC4569429; doi:10.1371/journal.pone.0138047)
Supplement: S1 File — (DOCX) [file pone.0138047.s005.docx]

**S1 Method: Mouse Body Weight and Plasma Triglycerides**

Mouse body weight was assessed weekly during the whole experimental period (10-32 weeks). Plasma triglyceride levels were measured using a commercial kit (BioAssay Systems, EGTA-200).

**S1 Fig: Mouse body weight and plasma levels of triglycerides.** A: Body weight was monitored on 32-weeks old apoE^-/-^ mice (n=10), CRD apoE^-/-^ mice (n=20) and CRD apoE^-/-^ mice treated with pitavastatin (CRD apoE^-/-^ PTV, n=20). Levels of triglycerides (B) were measured in plasma from apoE^-/-^ mice (n=8), CRD apoE^-/-^ mice (n=11) and CRD apoE^-/-^ mice treated with pitavastatin (CRD apoE^-/-^ PTV, n=17). Data are shown as mean ± SEM.
