# Supplementary material for: Pitavastatin Reduces Inflammation in Atherosclerotic Plaques in Apolipoprotein E-Deficient Mice with Late Stage Renal Disease
Source: PLoS One. 2015 Sep 14;10(9):e0138047. doi: 10.1371/journal.pone.0138047 (PMC4569429; doi:10.1371/journal.pone.0138047)
Supplement: S2 File — (DOCX) [file pone.0138047.s006.docx]

**S2 Method: Quantification of Calcium and Phosphate in Brachiocephalic Arteries**

10-μm sections were cut from frozen brachiocephalic arteries. Total 10 sections were collected from each arteries and dissolved in 100 μL of 0.5N HCl and incubated overnight on a rotation shaker. Levels of calcium and phosphate were measured using calcium colorimetric assay kit (BioVision, Catalog K380-250) and QuantiChrom Phosphate Assay Kit (BioAssay Systems, Catalog DIPI-500), respectively. The Spearman correlation between calcium and phosphate were analyzed by GraphPad Prism (Version 5.04).

**S2 Fig: Pitavastatin has no significant effect on the deposition of calcium and phosphate in brachiocephalic arteries.**  Quantitative measurement of the levels of calcium (A) and phosphate (B) in brachiocephalic arteries from apoE^-/-^ mice (n=5), CRD apoE^-/-^ mice (n=6) and CRD apoE^-/-^ mice treated with pitavastatin (CRD apoE^-/-^ PTV, n=6). Data are shown as mean ± SEM. C: Correlation between levels of calcium and phosphate quantified in brachiocephalic arteries (r=0.755, p=0.0005, n=17).
