# Supplementary material for: Pitavastatin Reduces Inflammation in Atherosclerotic Plaques in Apolipoprotein E-Deficient Mice with Late Stage Renal Disease
Source: PLoS One. 2015 Sep 14;10(9):e0138047. doi: 10.1371/journal.pone.0138047 (PMC4569429; doi:10.1371/journal.pone.0138047)
Supplement: S3 File — (DOCX) [file pone.0138047.s007.docx]

**S3 Method: Osteopontin Expression in Polarized M1 or M2 Macrophages**

Peritoneal macrophages were stimulated by 10 ng/mL of murine IFN-γ or 20 ng/mL murine IL-4 for 24 hours to induce M1 or M2 polarization, respectively. Levels of iNOS, TNF-α, MRC1 (Mannose Receptor, C Type 1), Arg1 (Arginase 1) and osteopontin mRNA were detected by real time-PCR and normalized by mRNA levels of GAPDH.

**S3 Fig: Osteopontin mRNA expression in M1 and M2 macrophages.** Mouse peritoneal macrophages were stimulated with IFN-γ and IL-4 to induce M1 and M2 polarization, respectively. Levels of iNOS (A), TNF-α (B), MRC1(C), Arg1(D) and osteopontin (E) gene expression were detected by real time-PCR and normalized by mRNA levels of GAPDH. Data are shown as mean ± SEM (n = 4 each group).
