# Supplementary material for: Pitavastatin Reduces Inflammation in Atherosclerotic Plaques in Apolipoprotein E-Deficient Mice with Late Stage Renal Disease
Source: PLoS One. 2015 Sep 14;10(9):e0138047. doi: 10.1371/journal.pone.0138047 (PMC4569429; doi:10.1371/journal.pone.0138047)
Supplement: S4 File — (DOCX) [file pone.0138047.s008.docx]

**S4 Method: Effect of NF-κB Activation Inhibitors on the Osteopontin Expression in Peritoneal Macrophages**

Mouse peritoneal macrophages were pre-incubated with either DMSO control or 3 μM Bay 11-7802 (Sigma Aldrich B5556) or 10 μM of JSH-23 (EMD Millipore 481408) for one hour, followed by adding 5 mM phosphate stimuli for another 12 hours. Levels of osteopontin mRNA were detected by real time-PCR and normalized by mRNA levels of GAPDH.

**S4 Fig: NF-κB activation inhibitors have no effect on the osteopontin mRNA expression induced by phosphate in peritoneal macrophages.** Mouse peritoneal macrophages were preincubated with DMSO, Bay 11-7802 or JSH-23 before stimulation with phosphate. Levels of osteopontin mRNA were detected by real time-PCR and normalized by mRNA levels of GAPDH. Data are shown as mean ± SEM (n=6 each group).
